# Data Sharing Under the General Data Protection Regulation: Time to Harmonize Law and Research Ethics?

**DOI:** 10.1161/HYPERTENSIONAHA.120.16340

**Published:** 2021-02-15

**Authors:** Antonia Vlahou, Dara Hallinan, Rolf Apweiler, Angel Argiles, Joachim Beige, Ariela Benigni, Rainer Bischoff, Peter C. Black, Franziska Boehm, Jocelyn Céraline, George P. Chrousos, Christian Delles, Pieter Evenepoel, Ivo Fridolin, Griet Glorieux, Alain J. van Gool, Isabel Heidegger, John P.A. Ioannidis, Joachim Jankowski, Vera Jankowski, Carmen Jeronimo, Ashish M. Kamat, Rosalinde Masereeuw, Gert Mayer, Harald Mischak, Alberto Ortiz, Giuseppe Remuzzi, Peter Rossing, Joost P. Schanstra, Bernd J. Schmitz-Dräger, Goce Spasovski, Jan A. Staessen, Dimitrios Stamatialis, Peter Stenvinkel, Christoph Wanner, Stephen B. Williams, Faiez Zannad, Carmine Zoccali, Raymond Vanholder

**Affiliations:** 1 From the Systems Biology Center, Biomedical Research Foundation, Academy of Athens, Greece (A.V.); 2 FIZ Karlsruhe – Leibniz-Institut für Informationsinfrastruktur, Eggenstein-Leopoldshafen, Germany (D.H., F.B.); 3 European Molecular Biology Laboratory, European Bioinformatics Institute (EMBL-EBI), Wellcome Genome Campus, Hinxton, Cambridge, United Kingdom (R.A.); 4 SAS RD-Néphrologie and Bio-Communication Cardio-Métabolique (BC2M) EA7288 and University Hospital Lapeyronie, University of Montpellier, France (A.A.); 5 KfH-Nierenzentrum und Klinikum St. Georg, Nephrologie, Leipzig, Germany (J.B.); 6 Istituto di Ricerche Farmacologiche Mario Negri IRCCS, Bergamo, Italy (A.B., G.R.); 7 Department of Analytical Biochemistry, University of Groningen, The Netherlands (R.B.); 8 Vancouver Prostate Centre, Department of Urologic Sciences, University of British Columbia, Canada (P.C.B.); 9 Institute of Genetics and Molecular and Cellular Biology, Institut de cancérologie Strasbourg Europe, Université de Strasbourg, France (J.C.); 10 University Research Institute of Maternal and Child Health & Precision Medicine, National and Kapodistrian University of Athens, ‘Aghia Sophia’ Children’s Hospital, Greece; (G.P.C.); 11 Institute of Cardiovascular and Medical Sciences, University of Glasgow, United Kingdom (C.D.); 12 Laboratory of Nephrology, Department of Immunology and Microbiology, Leuven, Belgium (P.E.); 13 Department of Health Technologies, Tallinn University of Technology, Estonia (I.F.); 14 Nephrology Section, Department of Internal Medicine and Pediatrics, Ghent University Hospital, Belgium (G.G., R.V.); 15 Translational Metabolic Laboratory, Department of Laboratory Medicine, Radboud Institute for Molecular Life Sciences, Radboud University Medical Center, Nijmegen, The Netherlands (A.J.v.G.); 16 Department of Urology, Medizinische Universität Innsbruck, Austria (I.H.); 17 Departments of Medicine and of Epidemiology and Population Health and Meta-Research Innovation Center at Stanford (METRICS), Stanford University (J.P.A.I.); 18 Institute of Cardiovascular Research, RWTH Aachen University, Germany (J.J., V.J.); 19 Cancer Biology and Epigenetics Group, Portuguese Oncology Institute of Porto and Abel Salazar Institute of Biomedical Sciences, University of Porto, Portugal (C.J.); 20 Division of Surgery, Department of Urology, The University of Texas MD Anderson Cancer Centre, Houston (A.K.); 21 Div. Pharmacology, Utrecht Institute for Pharmaceutical Sciences, Utrecht University, NL (R.M.); 22 Department of Internal Medicine IV (Nephrology and Hypertension), Medizinische Universität Innsbruck, Austria (G.M.); 23 Mosaiques Diagnostics and Therapeutics AG, Hannover, Germany (H.M.); 24 Department of Nephrology and Hypertension, IIS – Fundación Jiménez Díaz-UAM, Madrid, Spain (A.O.); 25 Steno Diabetes Center, University of Copenhagen, Denmark (P.R.); 26 Institut National de la Santé et de la Recherche Médicale (INSERM), U1048, Institut of Cardiovascular and Metabolic Disease, Toulouse and Université Toulouse III Paul-Sabatier, France (J.P.S.); 27 Urologie 24, Nuremberg, and Department of Urology, Friedrich-Alexander University of Erlangen, Germany (B.J.S-D).; 28 Department of Nephrology, University Clinical Center Skopje, North Macedonia (G.S.); 29 Research Institute Alliance for the Promotion of Preventive Medicine, Mechelen, Belgium, Biomedical Science Group, University of Leuven (J.A.S.); 30 Bioartificial organs, Department of Biomaterials Science and Technology, Technical Medical Centre, University of Twente, Enschede, The Netherlands (D.S.); 31 Department of Renal Medicine M99, Karolinska University Hospital, Stockholm, Sweden (P.S.); 32 Department of Medicine, Division of Nephrology, University Hospital, Würzburg, Germany (C.W.); 33 Department of Surgery, Division of Urology, The University of Texas Medical Branch, Galveston (S.B.W.); 34 Centre d’Investigation Clinique Inserm and Université de Lorraine, CHU Nancy, France (F.Z.); 35 Clinical Epidemiology and Physiopathology of Renal Diseases and Hypertension of Reggio Calabria, National Council of Research, Institute of Clinical Physiology, Italy (C.Z.); 36 European Kidney Health Alliance (EKHA), Brussels, Belgium (R.V.).

**Keywords:** biomedical research, data management, Ethics, Research, Government Regulation, informed consent

## Abstract

The General Data Protection Regulation (GDPR) became binding law in the European Union Member States in 2018, as a step toward harmonizing personal data protection legislation in the European Union. The Regulation governs almost all types of personal data processing, hence, also, those pertaining to biomedical research. The purpose of this article is to highlight the main practical issues related to data and biological sample sharing that biomedical researchers face regularly, and to specify how these are addressed in the context of GDPR, after consulting with ethics/legal experts. We identify areas in which clarifications of the GDPR are needed, particularly those related to consent requirements by study participants. Amendments should target the following: (1) restricting exceptions based on national laws and increasing harmonization, (2) confirming the concept of broad consent, and (3) defining a roadmap for secondary use of data. These changes will be achieved by acknowledged learned societies in the field taking the lead in preparing a document giving guidance for the optimal interpretation of the GDPR, which will be finalized following a period of commenting by a broad multistakeholder audience. In parallel, promoting engagement and education of the public in the relevant issues (such as different consent types or residual risk for re-identification), on both local/national and international levels, is considered critical for advancement. We hope that this article will open this broad discussion involving all major stakeholders, toward optimizing the GDPR and allowing a harmonized transnational research approach.

## Abstract 2

Supplemental Digital Content is available in the text.

The General Data Protection Regulation (GDPR) became binding law in all European Union (EU) Member States in May 2018.^1,2^ As a Regulation, it is, in principle, directly applicable to all EU Member States, superseding existing Member State laws. It, thus, represents a significant step toward harmonizing EU data protection laws.^3^

The GDPR applies to the processing of personal data across a large range of contexts, including those used in biomedical research and pertaining to vast areas of translational and clinical research activities, although it was probably not drafted with all the latter purposes in mind. As such, it has instigated multiple discussions among researchers and, in several cases, has raised major concerns, particularly, but not exclusively, in the genomics community (reviewed in study by Townend,^3^ Phillips,^4^ and Hallinan^5^).

With the present intense need for international scientific data and bio-sample sharing, actively supported by major publishers and research funders,^6,7^ GDPR requirements, particularly with respect to specific consent for data and bio-sample sharing, generate confusion and uncertainty and create conflicts between Regulation and Research Ethics.^8^ Under the fear of legal and social sanctions in combination with the threat of huge penalties as a consequence of violating GDPR, scientists have become reluctant to exchange data and bio-samples for secondary research.^9^ Increasingly, data and bio-sample use is restricted (to eg, specific hospitals, research institutions, or regions). In addition, the basic principle of scientific publishing that all relevant research data must be made accessible to ensure transparency and data reuse is frequently violated. Public health emergencies (such as the Ebola and Zika virus outbreaks and especially the ongoing coronavirus disease 2019 [COVID-19] pandemic) exemplify the need to facilitate swift and safe data sharing around the globe, without legal delays.^10,11^

This article aims to review the main principles of GDPR, in particular, pertinent to consent, as related to biomedical research; in this context, it discusses the practical problems related to data and bio-sample sharing, currently encountered by researchers, perceived to be linked directly or indirectly to GDPR; it also provides suggestions for interpretations and potential adaptations of the Regulation to better fit the practical realities of current biomedical research.

## Consent and Personal Data in the GDPR

Focusing on the most relevant issues, basic definitions and major aspects of the Regulation with direct impact on biomedical research are summarized in Table 1.

**Table 1.**
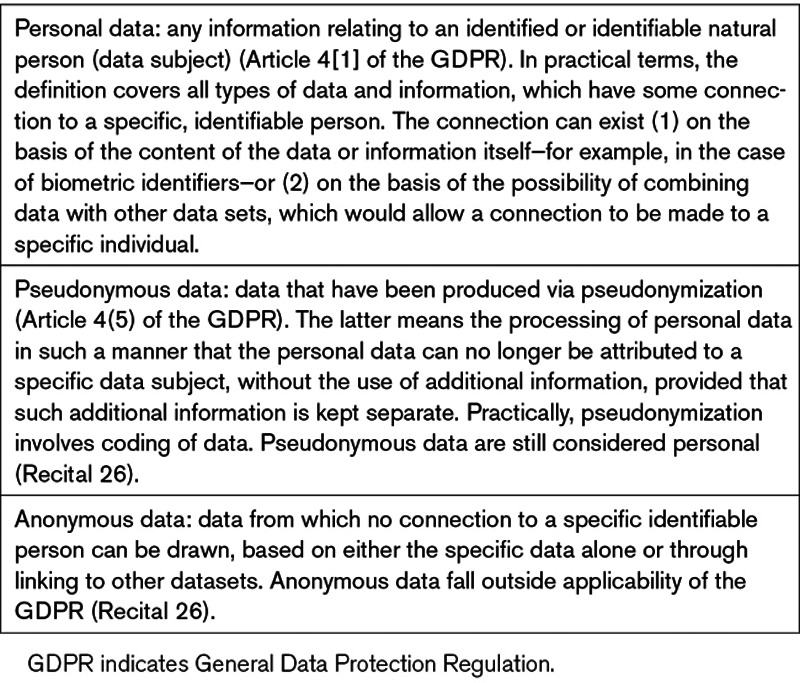
Basic GDPR Definitions Relevant to Personal Data

Processing of personal data concerning health is a priori prohibited (Article 9). Exemptions can be made based on consent which, as shown (Table 1), involves a clear, affirmative action in GDPR (Article 4.11). This action cannot simply be assumed based on failure to opt-out (eg, by not refusing preticked boxes or default settings). In addition, the scope of consent covers data processing for one or more specific purposes (Article 6). “Specific” is not explicitly defined but interpretations suggest that “the objectives of research, the principal investigator and the project’s duration are specified”.^12^

Exceptions allowing research without consent exist, based on Member State law for “archiving purposes in the public interest, scientific or historical research purposes” (Articles 6, 9). Alternatively, there is the possibility of obtaining consent for purposes not fully specified in advance (what we would call “broad” consent), related to “certain areas of scientific research, when in keeping with recognized ethical standards” (Recital 33). Nevertheless, there is confusion about the extent for which these exceptions could be applicable.^13^

Both directly identifiable and pseudonymized data used by researchers should be treated as personal data (Table 1), based on the presumed residual risk of research subject identification, in case of pseudonymization (summarized in study by Rumbold and Pierscionek^14^). Anonymized data are not affected or regulated by GDPR. To ensure anonymization, simply changing the identity label is insufficient. Multiple techniques are existent for further data perturbation to avoid (re) identification based on the variables listed.^15^

## Current Status in Biomedical Research Data and Bio-Sample Sharing

Sharing is an undisputed ethical obligation of any investigator (including those conducting patient-centered research) and is a prerequisite for scientific and clinical advancement.^16^ To frame this basic condition, pertinent ethical norms exist, including study monitoring by independent ethics committees, aiming to protect human subjects, while at the same time, promoting research collaboration (for a historical perspective, see study by Phillips^4^). The need for sharing in research becomes even more obvious and critical, when studying rare diseases (such as rare cardiac diseases^17^), which requires compilation of resources to allow meaningful research. In addition, and regardless of the disease incidence rates, the increasing diversity and complexity of molecular profiles and pathophysiologic phenotypes, underscores the need for adopting a systems integrative approach to define associations and causes, and, ultimately, biology-driven biomarkers and therapeutic targets. As an example, meta-analysis of genome-wide association datasets enables the identification of cardiovascular or kidney disease risk factors or confounders, that are too rare to be revealed at the individual study level.^18,19^ Likewise, fueling artificial intelligence algorithms with sufficiently large, multi-level datasets (clinical, pathophysiological, imaging, molecular), offers the unique opportunity to understand disease in a holistic manner.^20,21^ Along the same lines, data linkage can lead to better disease predictors, which was recently shown of particular value in stroke research.^22^ Such large scale holistic analyses can only be realized via data sharing and data reuse and are actively supported by major medical associations (eg reflected in the establishment of standards for data collection by the American Heart Association/American College of Cardiology Task Force on Data Standards^21^) and research funders, such as the EU Framework Program Horizon 2020, the National Institutes of Health, United States, and others.

GDPR-triggered restrictions linked to the requirements for specific consent generate uncertainty and slow down or even prohibit research activities. Contributing to this problem are the exponential developments in molecular technologies, which allow high resolution multidimensional analyses of a biological sample, making it hard or even impossible in the case of high-resolution genetic information, to truly anonymize study participants.^13,23^ Having to comply with an extensive (88 pages) legal framework increases the need for legal advice. As a result, collectively, valuable data and clinical samples may remain unused or significantly underused, causing a decrease of the power of the analyses.

To receive guidance on this critical issue of data and bio-sample use and sharing, as linked to study participant consent requirements in the context of the GDPR, several relevant translational networks (eg, the European Uremic Toxin Work Group [EuTox—https://www.uremic-toxins.org/], the European Society of Urologic Research [ESUR; https://uroweb.org/section/esur/information/], the Clinical Biomarkers COST Action [CliniMark; https://clinimark.eu/], and the International Bladder Cancer Network [IBCN; http://ibcnweb.net/]), initiated a discussion on the topic.

## Reflections on Implementing GDPR in Scientific Data and Sample Sharing

The main questions/problems currently distressing researchers and respective answers deduced from the Regulation and discussions with legal experts are summarized in Table 2.

**Table 2.**
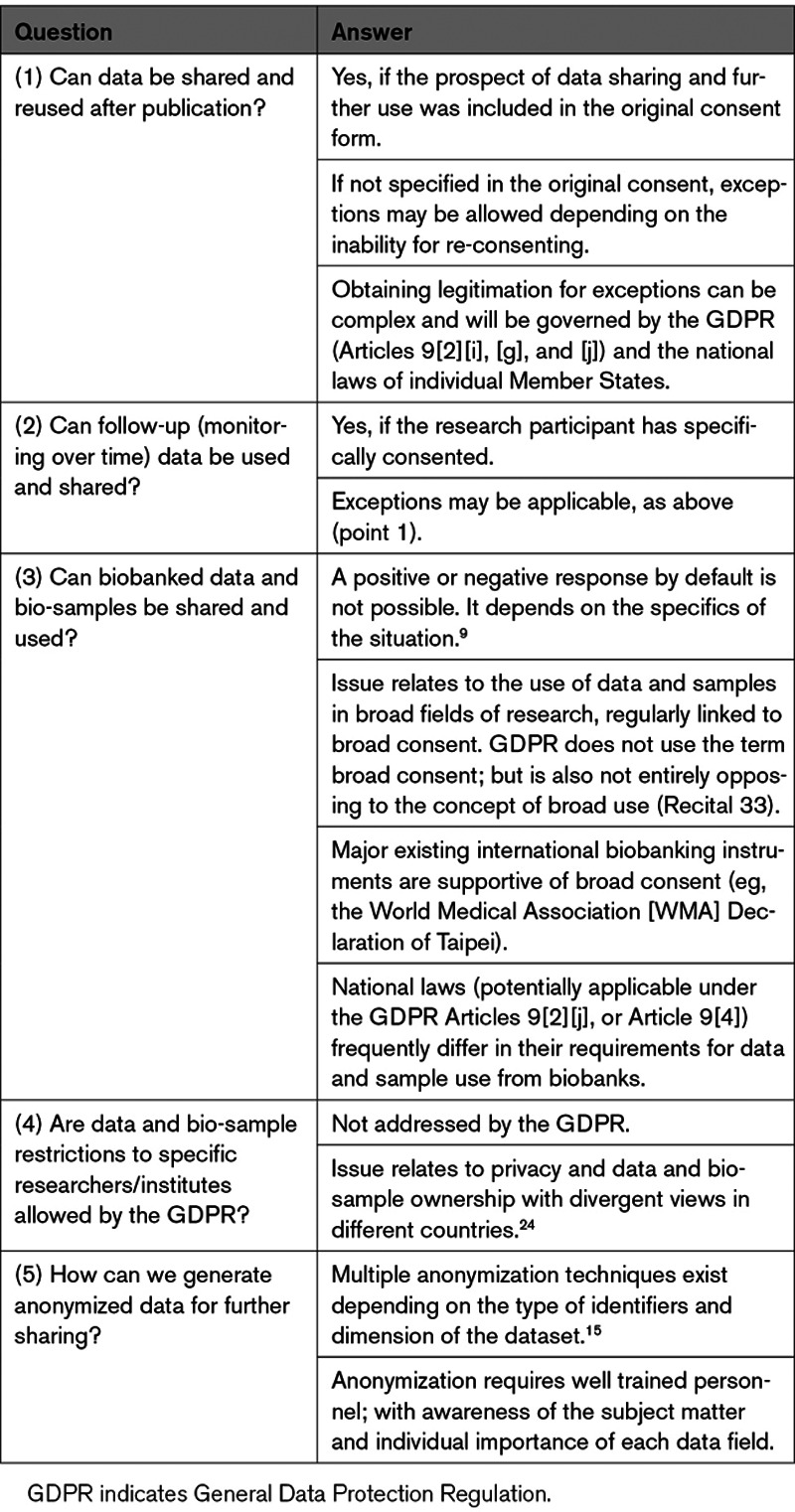
Main Practical Questions-Concerns of Researchers and Respective Answers Deduced From the GDPR

The answers provided (Table 2) highlight some main impediments perceived to be directly or indirectly imposed by the GDPR on biomedical research data and bio-sample sharing:

(1) Although the initial aim of the GDPR was to harmonize practices in the EU Member States, hence facilitating data sharing, a significant part of decision-making is still left to the Member States, increasing confusion and bureaucratic complexity. As a consequence, implementation of multinational collaborative projects relying on data and bio-sample sharing and mobility frequently face de facto regulatory deadlocks, which in our experience, to the least, slow down progress. Even more restrictions apply in the case of research involving partners outside the EU (including collaborations with European countries, Japan, or the United States), requiring a “lawful basis for making the transfer”.^9^

(3) Consent to data use ensuring protection of participants, undoubtedly constitutes a pillar of clinical research ethics and the principal condition to obtain ethical approval. However, the stated need for specific consent raises doubts as to the legitimacy of the use of valuable stored data and bio-samples covered by pre-GDPR consent forms. Calling for national regulation to legitimate processing, when the specific consent is not fully available or cannot be readily obtained, opens Pandora’s box in terms of the complexity of the legal landscape. The high complexity in relation to the scope of consent is illustrated by the problems that arise when existing data and bio-samples collected with the purpose of identifying specific biomarkers, for instance, for cardiovascular disease, would be considered as control in a study targeting the identification of specific chronic kidney disease biomarkers. In the absence of an explicit specification of this latter prospect in the original consent referring to cardiovascular disease, the use of such data and samples in chronic kidney disease research may be, a priori, prohibited. A possibility may exist to allow reuse with a supplemental consent. If this is not possible, reuse may be possible if a relevant national law has been enacted. However, in this latter case, use of Member State law to legitimate processing may end up causing issues in cases of collaborations requiring cross-border data and bio-sample exchange involving multiple EU Member States, as national laws do not always align.

(3) A potential solution in the latter case could be the use of data and bio-samples in an anonymized manner, hence, being exempt from GDPR and (re) consent requirements. However, considering the frequent availability of multiparametric, clinical, pathological, and molecular data characterizing a study participant, as mentioned above, it is reasonable to assume that true anonymization is only possible at the cost of significant loss of information (perturbance of data), especially in the context of -omics and Big Data analyses.^13,23^ Furthermore, anonymization may be incompatible with many current research objectives (such as study of disease progression in a longitudinal, individualized manner, as required to establish treatment in a personalized approach).

## Solutions

Specification of the impediments forms the basis for a fruitful discussion toward resolution or improvement. Potential ways forward might include (summarized in Table 3) the following:

**Table 3.**
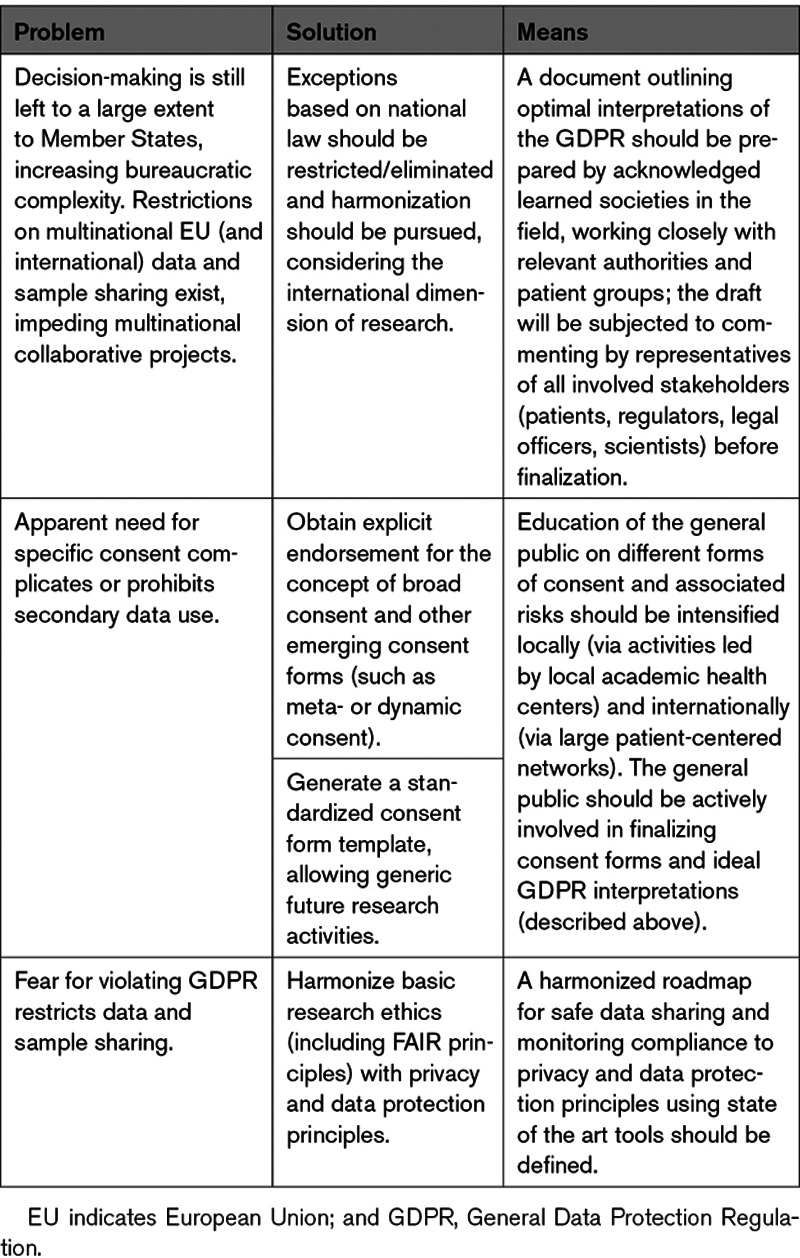
Summary of Main Problems, Suggested Solutions, and Means to Achieve Them

(1) Similar to other areas in medicine,^25^ public/general population education and engagement in research pipelines is critical. This would target getting a clear understanding of the definitions and associated benefits and risks with broad and other emerging forms of consent, namely meta-consent (allowing the research participant to define what type, when and how consent may be provided in the future^26^) or dynamic consent (through web platforms^27^). In addition, it would explain residual risks for reidentification. There is an apparent ever increasing willingness of individuals to share their data for research advancement, reflected in the existence of large personal genome projects (such as the Harvard or Canadian, United Kingdom, Austrian, Korean, and other national personal genome projects; https://www.personalgenomes.org/). Interactions of researchers and patients should be intensified in the form of large patient-linked networks (for example, the European Kidney Health Alliance; involving Kidney Patients and Foundations, Nephrologists and Nurses, http://ekha.eu/; or the US National Patient-Centered Clinical Research Network [PCORnet]), consisting of health care centers, as well as patient organizations (Patient-Powered Research Networks; https://pcornet.org/patient-centric/), and via regular local outreach initiatives led by academic health centers, with the aim of achieving patient-centered informed consent, anticipating as much as possible future developments.

(2) Addressing issues related to secondary use of data and need for respective re-consent is the most challenging problem, as, for the time being, this seems to be in many cases subject to national law. In our opinion, this cannot be solved by an ad hoc administrative, legal, or policy change only; it must be the topic of a broad multidisciplinary discussion involving researchers, patient advocacy groups and the general public, ethics experts, regulators, and politicians targeting clarity and harmonization, as much as possible going beyond national boundaries, in line with the international character of high-level research. An example in this direction forms the design of the Final Rule, the latest update of the federal policy for the protection of human subjects in the United States, which emerged after being subject to commenting by the general public, various agencies, and expert stakeholders.^28^ Our proposal is that the lead to open this discussion is taken by a Consortium of several acknowledged societies involved in the field, including patient advocacy groups; these should work closely with relevant authorities at the EU level (EU Directorate General [DG] for Research and Innovation, the European Commissioner for Research, Innovation and Science, and the Committee of Members of European Parliament for Industry, Research and Energy), and eventually also involve the respective national authorities, toward harmonizing interpretations of the law, aiming at minimizing boundaries impeding cross-border research in the EU.

(3) In this context, the generation of a standardized consent form (broad consent allowing generic future research activities), acceptable to all stakeholders involved, would be of enormous benefit. Such efforts in the case of biobanking do exist, for example, in Germany,^12^ but should be expanded on a harmonized European (and, possibly, international) level. Such a form should inform research participants that complete anonymization may not always be feasible (eg, in the case of whole genome sequencing) and that pseudonymization will be applied to minimize risk for reidentification. As a step in this direction, a template consent form, using a multi-omics study as a test case, is provided (Data Supplement). This is adapted from, and borrows text from existing consent forms (Health Innovation and Research Institute from the Ghent University Hospital, Belgium^29^; and Human Cancer Models Initiative, United States^30^). This form is intended to serve as a basis for discussion with ethics/legal experts and research participants-patient representatives for further improvement (including abbreviating and simplifying to obtain a legally and ethically sound template, that meets the needs of both research participants and researchers).

(4) The frequently observed restriction of data and bio-samples to specific institutions or countries, although not instructed by GDPR, is often the consequence of fear of violating GDPR. Basic research ethics through implementation of FAIR principles (Findable, Accessible, Interoperable, and Reusable data^16^) and compliance with privacy and data protection principles should be observed and can be monitored, as recently described.^31^ Systems, such as DATAshield, for simultaneous analysis of multi-source individual data without actual data transfer,^32^ data hubs allowing highly regulated access,^3^ or blockchain platforms offering de-centralised data access and interoperability,^33^ provide data sharing solutions which help maintaining data integrity and patient security. Both the patient and scientific communities would benefit from a harmonized European roadmap on this issue.

## Conclusions

The collective experience accrued by now renders the time ripe to assess whether or not the GDPR is on the right track with respect to scientific data and bio-sample sharing and facilitating adherence to the basic research ethics of data sharing. Although, in principle, the GDPR is a step toward regulatory harmonization, based on the issues presented above, adjustments are urgently needed. These should (1) increase harmonization by minimizing differences caused by exceptions based on national laws; (2) seek explicit endorsement of the concept of broad consent and consequently; (3) better define the roadmap for secondary use of data and bio-samples at European (and, if possible, international) level. The lead to formulate the working draft should be taken by a team of acknowledged learned societies in the field including patient advocacy groups working closely with experts at the EU level; finalization should be made following a period of commenting by a broad multi-stakeholder audience. This process should evolve in parallel to promoting engagement and education of the public in the relevant definitions (of, eg, broad [specific, meta-, or dynamic] consent; data sharing; residual risk for re-identification), led by academic health centers on a local level and amplified by large patient-centered multidisciplinary networks. We hope that this article will serve as a catalyst for this broad discussion involving all major stakeholders, toward optimizing GDPR to facilitate biomedical research and to produce social benefit and welfare.

## Sources of Funding

None.

## Disclosures

None.

## Supplementary Material



## References

[1] Regulation (EU) 2016/679 of the European Parliament and of the Council of 27 April 2016 on the protection of natural persons with regard to the processing of personal data and on the free movement of such data, and repealing Directive 95/46/EC (General Data Protection Regulation). Official Journal of the European Union L 119/1. Accessed September 25, 2020. https://eur-lex.europa.eu/legal-content/EN/TXT/PDF/?uri=CELEX:32016R0679&from=EN.

[2] Bentzen HB Høstmælingen N. Balancing protection and free movement of personal data: the new European Union General Data Protection Regulation. Ann Intern Med. 2019; 170:335–337. doi: 10.7326/M18-2782 30776801 10.7326/M18-2782

[3] Townend D. Conclusion: harmonisation in genomic and health data sharing for research: an impossible dream? Hum Genet. 2018; 137:657–664. doi: 10.1007/s00439-018-1924-x 30120573 10.1007/s00439-018-1924-x PMC6132652

[4] Phillips M. International data-sharing norms: from the OECD to the general data protection regulation (GDPR). Hum Genet. 2018; 137:575–582. doi: 10.1007/s00439-018-1919-7 30069638 10.1007/s00439-018-1919-7 PMC6132662

[5] Hallinan D. Broad consent under the GDPR: an optimistic perspective on a bright future. Life Sci Soc Policy. 2020; 16:1. doi: 10.1186/s40504-019-0096-3 31903508 10.1186/s40504-019-0096-3 PMC6943899

[6] Piwowar HA Becich MJ Bilofsky H Crowley RS; caBIG Data Sharing and Intellectual Capital Workspace. Towards a data sharing culture: recommendations for leadership from academic health centers. PLoS Med. 2008; 5:e183. doi: 10.1371/journal.pmed.0050183 18767901 10.1371/journal.pmed.0050183 PMC2528049

[7] Bloom T Ganley E Winker M. Data access for the open access literature: PLOS’s data policy. PLOS Med. 2014; 11:e1001607. doi: 10.1371/journal.pmed.1001607

[8] Mascalzoni D Bentzen HB Budin-Ljøsne I Bygrave LA Bell J Dove ES Fuchsberger C Hveem K Mayrhofer MT Meraviglia V. Are requirements to deposit data in research repositories compatible with the European Union’s General Data Protection Regulation? Ann Intern Med. 2019; 170:332–334. doi: 10.7326/M18-2854 30776795 10.7326/M18-2854

[9] Peloquin D DiMaio M Bierer B Barnes M. Disruptive and avoidable: GDPR challenges to secondary research uses of data. Eur J Hum Genet. 2020; 28:697–705. doi: 10.1038/s41431-020-0596-x 32123329 10.1038/s41431-020-0596-x PMC7411058

[10] Chretien JP Rivers CM Johansson MA. Make data sharing routine to prepare for public health emergencies. PLoS Med. 2016; 13:e1002109. doi: 10.1371/journal.pmed.1002109 27529422 10.1371/journal.pmed.1002109 PMC4987038

[11] Wigginton NS Cunningham RM Katz RH Lidstrom ME Moler KA Wirtz D Zuber MT. Moving academic research forward during COVID-19. Science. 2020; 368:1190–1192. doi: 10.1126/science.abc5599 32467332 10.1126/science.abc5599

[12] Strech D Bein S Brumhard M Eisenmenger W Glinicke C Herbst T Jahns R von Kielmansegg S Schmidt G Taupitz J. A template for broad consent in biobank research. Results and explanation of an evidence and consensus-based development process. Eur J Med Genet. 2016; 59:295–309. doi: 10.1016/j.ejmg.2016.04.002 27130428 10.1016/j.ejmg.2016.04.002

[13] Price WN 2nd Kaminski ME Minssen T Spector-Bagdady K. Shadow health records meet new data privacy laws. Science. 2019; 363:448–450. doi: 10.1126/science.aav5133 30705168 10.1126/science.aav5133 PMC6417878

[14] Rumbold JM Pierscionek B. The effect of the general data protection regulation on medical research. J Med Internet Res. 2017; 19:e47. doi: 10.2196/jmir.7108 28235748 10.2196/jmir.7108 PMC5346164

[15] . UK Care Quality Commission- 20150327 Anonymisation Guidance V1.0. Accessed September 25, 2020. https://www.cqc.org.uk/sites/default/files/Anonymisation%20Guidance.pdf.

[16] Wilkinson MD Dumontier M Aalbersberg IJ Appleton G Axton M Baak A Blomberg N Boiten JW da Silva Santos LB Bourne PE. The FAIR Guiding Principles for scientific data management and stewardship. Sci Data. 2016; 3:160018. doi: 10.1038/sdata.2016.18 26978244 10.1038/sdata.2016.18 PMC4792175

[17] Adler A Kirchmeier P Reinhard J Brauner B Dunger I Fobo G Frishman G Montrone C Mewes HW Arnold M. PhenoDis: a comprehensive database for phenotypic characterization of rare cardiac diseases. Orphanet J Rare Dis. 2018; 13:22. doi: 10.1186/s13023-018-0765-y 29370821 10.1186/s13023-018-0765-y PMC5785853

[18] Sampson MG Kang HM. Using and producing publicly available genomic data to accelerate discovery in nephrology. Nat Rev Nephrol. 2019; 15:523–524. doi: 10.1038/s41581-019-0166-z 31182850 10.1038/s41581-019-0166-z

[19] Leon-Mimila P Wang J Huertas-Vazquez A. Relevance of multi-omics studies in cardiovascular diseases. Front Cardiovasc Med. 2019; 6:91. doi: 10.3389/fcvm.2019.00091 31380393 10.3389/fcvm.2019.00091 PMC6656333

[20] Kagiyama N Shrestha S Farjo PD Sengupta PP. Artificial intelligence: practical primer for clinical research in cardiovascular disease. J Am Heart Assoc. 2019; 8:e012788. doi: 10.1161/JAHA.119.012788 31450991 10.1161/JAHA.119.012788 PMC6755846

[21] Weintraub WS. Role of big data in cardiovascular research. J Am Heart Assoc. 2019; 8:e012791. doi: 10.1161/JAHA.119.012791 31293194 10.1161/JAHA.119.012791 PMC6662116

[22] Ung D Kim J Thrift AG Cadilhac DA Andrew NE Sundararajan V Kapral MK Reeves M Kilkenny MF. Promising use of big data to increase the efficiency and comprehensiveness of stroke outcomes research. Stroke. 2019; 50:1302–1309. doi: 10.1161/STROKEAHA.118.020372 31009352 10.1161/STROKEAHA.118.020372

[23] Rocher L Hendrickx JM de Montjoye YA. Estimating the success of re-identifications in incomplete datasets using generative models. Nat Commun. 2019; 10:3069. doi: 10.1038/s41467-019-10933-3 31337762 10.1038/s41467-019-10933-3 PMC6650473

[24] Kaye J Briceño ML Curren L Bell J Mitchell C Soini S Hoppe N Øien M Rial-Sebbag E. Consent for biobanking: the legal frameworks of countries in the BioSHaRE-EU project. Biopreserv Biobank. 2016; 14:195–200. doi: 10.1089/bio.2015.0123 27145287 10.1089/bio.2015.0123 PMC5967579

[25] Rao JK. Engaging public health in end-of-life issues: it is time to step up to the plate. Ann Intern Med. 2015; 162:230–231. doi: 10.7326/M14-2479 25486456 10.7326/M14-2479

[26] Ploug T Holm S. Meta consent - a flexible solution to the problem of secondary use of health data. Bioethics. 2016; 30:721–732. doi: 10.1111/bioe.12286 27628305 10.1111/bioe.12286 PMC5108479

[27] Budin-Ljøsne I Teare HJA Kaye J Beck S Bentzen HB Caenazzo L Collett C D’Abramo F Felzmann H Finlay T. Dynamic consent: a potential solution to some of the challenges of modern biomedical research. BMC Med Ethics. 2017; 18:4. doi: 10.1186/s12910-016-0162-9 28122615 10.1186/s12910-016-0162-9 PMC5264333

[28] . Rules and Regulations Federal Register 2017, 82, (12). https://www.govinfo.gov/content/pkg/FR-2017-01-19/pdf/2017-01058.pdf. Accessed September 25, 2020.

[29] . Biobank of Health Innovation and Research Institute, Belgium. http://hiruz.be/service/hb/. Accessed December 10, 2020.

[30] Informed Consent Template for HCMI. 2018:1–6. https://ocg.cancer.gov/sites/default/files/CMDC_Informed_Consent_Template_v2.pdf. Accessed December 10, 2020.

[31] Di Iorio CT Carinci F Oderkirk J Smith D Siano M de Marco DA de Lusignan S Hamalainen P Benedetti MM. Assessing data protection and governance in health information systems: a novel methodology of privacy and ethics impact and performance assessment (PEIPA) J Med Ethics. 2020. doi: 10.1136/medethics-2019-105948 10.1136/medethics-2019-105948 32220868

[32] Gaye A Marcon Y Isaeva J LaFlamme P Turner A Jones EM Minion J Boyd AW Newby CJ Nuotio ML. DataSHIELD: taking the analysis to the data, not the data to the analysis. Int J Epidemiol. 2014; 43:1929–1944. doi: 10.1093/ije/dyu188 25261970 10.1093/ije/dyu188 PMC4276062

[33] Hasselgren A Kralevska K Gligoroski D Pedersen SA Faxvaag A. Blockchain in healthcare and health sciences-A scoping review. Int J Med Inform. 2020; 134:104040. doi: 10.1016/j.ijmedinf.2019.104040 31865055 10.1016/j.ijmedinf.2019.104040

