# Supplementary material for: Data Sharing Under the General Data Protection Regulation: Time to Harmonize Law and Research Ethics?
Source: Hypertension. 2021 Feb 15;77(4):1029–35. doi: 10.1161/HYPERTENSIONAHA.120.16340 (PMC7968961; doi:10.1161/HYPERTENSIONAHA.120.16340)
Supplement: Supplementary file 1 [file hyp-77-1029-s001.pdf]

DATA SUPPLEMENT

Data sharing under the General Data Protection Regulation: time to harmonize law and research ethics?

Authors: Antonia Vlahou, Dara Hallinan, Rolf Apweiler, Angel Argiles, Joachim Beige, Ariela Benigni, Rainer Bischoff, Peter C. Black, Franziska Boehm, Jocelyn Ceraline, George P. Chrousos, Christian Delles, Pieter Evenepoel, Ivo Fridolin, Griet Glorieux, Alain J. van Gool, Isabel Heidegger, John P.A. Ioannidis, Joachim Jankowski, Vera Jankowski, Carmen Jeronimo, Ashish Kamat, Roos Masereeuw, Gert Mayer, Harald Mischak, Alberto Ortiz, Giuseppe Remuzzi, Peter Rossing, Joost P. Schanstra, Bernd J. Schmitz-Dräger, Goce Spasovski, Jan A Staessen, Dimitrios Stamatialis, Peter Stenvinkel, Christoph Wanner, Stephen B Williams, Faiez Zannad, Carmine Zoccali, Raymond Vanholder

Short title: Scientific Data Sharing: Legal under GDPR?

Corresponding Author:
Antonia Vlahou, Ph.D
Biomedical Research Foundation, Academy of Athens
Tel: +30 2106597506
Email: vlahoua@bioacademy.gr

representatives of participants

[Biological sample] collection in the context of research in [disease]

Title of the project:

Dear,

You/your child are/is invited to participate in a research project entitled [name of project]

Your participation/your child's participation is entirely **voluntary**. You may choose not to volunteer. This will not affect your care in any way.

You will not benefit from taking part in the project. The research is at an early stage, so it may only give us knowledge that will help people in the future.

Before you decide to take part in this project, **allow enough time to read** this information letter carefully **and discuss** it with the doctor, researcher or his/her representative, or with other people.

Please also take the time to **ask questions** if there are any unclear points or if you require additional information. It is important that you fully understand what your participation in this project involves. This process is called 'informed consent'.

Once you have decided to participate, **you will be asked to sign the consent form** at the back of this booklet. On this consent form you can make several choices, in order to reveal your specific wishes.

1. WHAT ARE THE OBJECTIVES OF RESEARCH?

This project targets the better understanding of mechanisms leading to [**disease**] and developing new biomarkers and therapies to **improve its clinical management**.

To reach this goal, the analysis of [**sample**] together with additional health, lifestyle and personal data of a large group of people/patients/study participants is required. The samples will be analyzed by researchers using new technologies that allow the **characterization of your genetic material (DNA) and other molecules (proteins, metabolites)** included in these samples. This produced information, is most useful when connected to **your clinical information, which includes information about you, your disease, how you were treated, and how you responded to that treatment**. This clinical information **will** therefore **be collected**, but all obvious identifiers (like your name, social security number, medical record number, address and phone number), will be removed when sharing your sample or data with researchers.

This sample collection is set up to serve research on [disease] as a primary goal. However, **your sample and collected data may also be used in the investigation of other human diseases** where it could serve as a valuable control to identify disease-specific mechanisms and/or therapies and/or biomarkers. Therefore, the objectives of the research are multiple:

- The samples and data will be used to better understand the interaction between genes, proteins, environment and lifestyle that influence the health status and development of [disease] as well as other human diseases.
- On the basis of this insight, efforts will be made to develop new therapeutics, treatments and tests in order to contribute to a general improvement in health care.
- This collection and generated data will allow many researchers to carry out high-quality scientific research towards better patient welfare in [disease] and other human diseases.

Your participation/your child's participation in this project means that you give "**broad consent**" to the use of your/your child's data and samples. This means that **you allow your/your child's personal and health information as well as your/your child's samples to be used for the specific project but also future scientific research in multiple studies** targeting better patient welfare and treatment in [disease] as well as other human diseases. At this moment, all specific studies in which the material and data will be used cannot be specified in detail. **The studies will always be pre-approved by an independent Ethical Committee.**

For certain additional studies, we may update your/your child's administrative and health data. You can indicate on the consent form whether you agree to this.

2. HOW MANY TEST SUBJECTS WILL PARTICIPATE IN THIS PROJECT AND WHY IS MY PARTICIPATION REQUESTED?

A total of [number of people] will participate in the project, within the age group [age range]

You/your child are/is invited to participate in this project because **you/your child are/is a suitable study participant** or a patient at [hospital] where specific research is conducted within the domain of [disease name].

You have had a clinical exam that identified [disease]. You are being asked to participate in this project because, as part of your planned medical procedure you are having [sample collection]. Normally, any extra [sample] not used by your physician for the diagnosis of [disease] would be thrown away.

3. WHAT IS REQUESTED?

We are requesting your **permission to use some of that extra [sample] collected during your visit to the [hospital]** not used by your physician for the diagnosis of [disease]. In addition, we ask **if you could donate [sample] for scientific purposes**. This will help scientists characterize [disease] in a more spherical manner. We also **request your permission to collect information from your medical records** to try to build a model of your [disease].

We will also collect data monitoring the outcome of your/your child's disease over time (number of years). These data will be stored pseudonymized (coded after removing personal identifiers such as name, address, date of birth-described also below-part 5), in the database to which only authorized staff members of [department name] have access.

4. WHAT DOES PARTICIPATION IN THE PROJECT MEAN TO YOU-ABILITY TO WITHDRAW

Participation in this research project **does not bring any immediate therapeutic benefit to you/your child**. However, your/your child's participation in this project may **contribute** to increased scientific knowledge about [disease] and other human diseases and may lead **to better treatment of future patients**.

You can choose which of the requested [sample] you voluntarily donate to this project by specifying this on the consent form. By [sample] we mean what you have voluntarily donated for scientific research, as well as material that you have donated for diagnostic or therapeutic purposes.

If you choose to participate/allow your child to participate in this research project, it is very important that you **answer as truthfully as possible the questions asked** by the doctor and the questionnaires given. **If** you/your child are/is **uncomfortable with certain questions**, you can tell us and **you should not answer them** any further.

We cannot know how long it will take to work with your samples to successfully analyze their molecular profile. You are the 'owner' of your/your child's collected body material. This means that **you can always demand that your/your child's stored samples are destroyed** without you/your child having to give a reason for doing so and **without this in any way affecting your/your child's treatment** or the further relationship with the researcher or the attending physician. **This will also not have any negative influence on the quality of the care** and your/your child's further follow-up. To withdraw from the study, you can contact your primary contact point [name of principal investigator] within the context of this research project, as described below. After this, you will receive a letter confirming the request to destroy your/your child's samples and the accompanying data. After receiving this written confirmation, your/your child's stored body material will be destroyed. If your/your child's samples/data have already been used or are in use in an ongoing study, they cannot be withdrawn, but will not be made available for other scientific studies.

Participation in this project is completely **voluntary**. You have the right to ask questions about the possible and/or known risks of this project at any time.

5. WHERE DOES THE PROJECT TAKE PLACE?

This project takes place in [**name of institution**]. **It is expected that, in the future, it will also take place to additional research settings** (academia, research institutes, private companies), and include additional researchers, in order to accelerate research in the field of [disease], and other human diseases.

The [name of department) is responsible for the practical aspects such as data collection and the safe storage of samples and data. Your primary contact for this project is [name of PI] and contact details are [contact information].

The study was pre-approved by an independent Commission for Medical Ethics [name of committee]. This project is carried out according to the Guidelines for Good Clinical Practice (ICH/GCP) and the Helsinki Declaration on the Protection of People Participating in Clinical Studies. **Under no circumstances should you consider the approval of the study by the Commission for Medical Ethics as an incentive to participate in this project.**

The following sections describe how your [samples] and information will be collected and studied if you give us this permission.

6. PROCESSING OF YOUR SAMPLES

You/your child will be asked to donate [sample] as part of the study. In total approximately [volume of biological fluid, as applicable] will be taken. You/your child will also be asked to complete an accompanying questionnaire. If necessary, you/your child can also be asked to have these samples taken (periodically), for a period of (number of months/years).

The collected material will be processed in the laboratory of [name of department], and stored pseudonymized (under a random number code, after removing personal identifiers such as name, address, social security number, date of birth) in [name of department]. The sample will be partly analysed for its molecular content (DNA, protein, metabolites) by research staff of [name of department] working on better [disease] management. In brief, the genetic information from your sample will be obtained by a method called sequencing. Sequencing allows researchers to read the codes of instructions that are spelled out in your DNA and help them identify genetic changes that result due to the [disease] process or that may be associated with your [disease]. In parallel, analysis of the proteins as well as smaller molecules (metabolites) by novel technologies will be performed which will allow their identification and quantification. The resulting data will be shared with the scientific community through secured databases and scientific publications (described also below) to facilitate research advancement though collaborative research.

The remaining sample will be stored to further support current and future research. Within the framework of scientific collaborations, your/your child's samples can be sent to other research centers (universities, research institutions, private companies), for analysis of new markers and treatments for [disease], but also serve as valuable control to study new biomarkers and treatments for other diseases.

7. PROJECT RESULTS

Information from the analysis of your samples, together with your clinical information will be put into a central database, along with information from the other people who volunteered for this project. **These databases will be accessible by the internet to the research community**. All the information in this database will only be labeled with the random number code; **it will not include your name, social security number, medical record number, address and phone, or any other obvious identifier**. To access this database, any researcher and their institution will have to:

- Agree to never use the data to identify the donors of the materials,
- Agree to use the data only for research projects

General scientific findings and new insights will be made available as aggregated results in the form of a scientific publication on the following website https://www.ncbi.nlm.nih.gov/pubmed?otool=ibeuglib
This publication of findings will by no means contain any identifying data, but is important for the rapid dissemination of scientific findings within the research community.

8. PROTECTING YOUR PRIVACY AND KEEPING YOUR INFORMATION CONFIDENTIAL

In accordance with the [national law] and the General Data Protection Regulation (or GDPR) of 25 May 2018, **your/your child's privacy will be respected** and you will have access to the data collected. Any incorrect data can be corrected at your request.

If you volunteer to be part of the project, this signed consent form will be stored in a locked file that will be accessible only to authorized people involved in this project. Personal and health data collected during the project will not be passed on to third parties such as employers, insurance companies or family members, unless imposed by legal proceedings.

Your [samples] and information will be pseudonymized, **which means that all your obvious identifiers (like name, social security number, medical record number, address, phone number, and others) will be stripped away**. Your samples and information will only be labeled with a **random number code**. This makes your samples and data not directly identifiable, however, **via the code they still can be traced back to you.**

Dr. [Physician] at [Institution] will keep a link between that random number code and your ID in a secure database. Only authorized people who have specifically agreed to protect your identity will have access to that database, and the link will not be shared with anyone outside of [Institution]. Both personal data and data concerning your/your child's health will be processed and stored for at least [number of years]. If desired, the Data Protection Officer [contact details] can provide you with more information about the protection of your personal data.

9. ACCESS TO SAMPLES AND PSEUDONYMISED DATA

Researchers requesting access to the samples or data **will receive these samples and data pseudonymized, so that identification of your/your child person is not directly possible**.
Only research projects approved by a competent Ethics Committee can have access to your pseudonymized samples and/or data for scientific research. This Committee assesses whether the research projects falls within the scope of this project and the given approval of you/your child as a participant. The confidentiality of your/your child's data will be handled with the utmost care.

These research projects may be led by **researchers connected to an academic center, a healthcare institution, a non-profit organization, a public institution or a private/commercial company**. In the consent form you can further specify whether or not you allow such access to your samples and data.

It is also possible that your/your child's samples/data may be used in the context of approved projects **outside [geographical area of research**]. You can also indicate your preferences on this aspect on the consent form.

Researchers who obtain samples and/or data must sign contracts with a strict description of their access and use of these samples and data. These researchers are not allowed to further disseminate, transfer or use data or samples for purposes other than those described in this consent. Researchers should also agree not to attempt to re-identify participants and should also report if this should occur.

Transferred samples in approved projects should either be destroyed or returned to the (department) at the end of the project so that they can still be used for other approved studies.

Representatives of the client, auditors, the Commission for Medical Ethics and the competent authorities, all bound by professional secrecy, **have direct access to your/your child's medical files** in order to check the procedures of the research project, without violating confidentiality. This can only be done **within the limits permitted by the relevant laws**. By signing the consent form, after prior explanation, you agree to this access.

You have the right to complain about how your/your child's information is being handled to the [**national supervisory authority**] responsible for enforcing data protection legislation.

10. WHAT ARE THE RISKS OF PARTICIPATING IN THIS PROJECT?

There are some risks if you decide to participate in this research project:

<u>Physical risks</u>
There are very few physical risks associated with this project. There are possible **side effects from [sample collection]** including [mild pain, bleeding, bruising, infection…,as applicable].

<u>Risks if information about you is accidently released</u>
Keeping your information confidential is very important to us and we use many safety measures to protect that information. However, some of this information may still be traceable to you and **we cannot guarantee that your identity will never become known**. It is possible, for example, that there could be violations to the security of the computer systems used to store the link between the random number code and your name or other identifiers. It is also possible that, in the future, someone could compare information in our databases with information from you (or a relative) in another database and be able to identify you (or your relative). While we believe that the risks to you and your family are low if your identity became known, **we are unable to tell you exactly what all of the risks are**.

If your identity became known, here are some of the possible risks:
• There could be psychological or social risks associated with loss of privacy. For example, your genetic information could potentially be used in ways that could cause you or your family distress by revealing that you (or a relative) carry a genetic disease. This could lead to the denial of life insurance for you (or a relative).
• Patterns of genetic information are shared by relatives. If your identity became known, it is possible that the identity of your relatives could also become known.

• Patterns of genetic variation also can be used by law enforcement agencies to identify a person or his/her relatives.
• There may also be other privacy risks that we have not foreseen.

11. WHAT ARE THE EXPECTED BENEFITS OR COSTS OF PARTICIPATING IN THIS PROJECT?

Benefits of participating in this project
You will not personally benefit from this project; the research is at an early stage. The main reason you may want to participate is to help researchers and health professionals around the world to better understand the basic causes of [disease] and other diseases so that they can find better ways to prevent, detect, treat, and cure them.

In the very low chance that Individual scientific findings of potential interest to your/your child's general state of health are produced (referred to as 'incidental findings'), the importance of these findings will first be confirmed by [name of committee] of [name of hospital] and the Ethical Committee. If it is judged that these findings are scientifically validated, have a clinical relevance and actions can be taken (therapeutic actions or preventive actions), concerning your/your child's state of health, this can be reported back. If you agree to be informed, you can tick this on the consent form. Your/your child's attending physician will then discuss the findings with you.

Costs and payments to you
It will not cost you anything to participate in this project. You will not be paid to participate in this project.

The chance that you will be physically injured as a result of participating in this project is very small. However, if you are physically injured as a result of participating in this project, emergency medical treatment for your research-related injury will be provided to you at no cost.

Commercialization
Your medical information and samples, and any generated data will only be used for research. However, it is possible that some of the research using your samples could eventually lead to the invention of new diagnostic tests, new drugs, or other products that could be sold by companies. This development can be done by a university, hospital, commercial company or a partnership of these. This means that it would be possible for researchers and commercial companies to benefit financially from your donation. If this would happen, you will not get any part of the profits from those products.

**Contact details:**
Primary contact point (medical administrator):

Consent Form

By signing this consent form, I agree to participate/make my child participate in the research project and declare the following:

	Initials when agreeing
• I have read and understood the document "Information letter for the participants project" page 1 to 8 and got a copy of it.<br>o I have received explanations about the research project, its purpose and set-up and about what is expected of me/my child. I received an explanation about the possible risks and benefits.<br>o I was given the opportunity and enough time to ask questions about the project.	
• I understand that participation in this project is voluntary<br>o I understand that I may withdraw myself/my child from this project at any time without giving up a reason<br>o This will in no way affect my/ my child's further treatment.	
o I am aware that this project was approved by an independent Commission for Medical Ethics This project will be carried out according to the Guidelines for Good Clinical Practice (ICH/GCP) and the Helsinki Declaration, drawn up to protect people participating in experiments.<br>o This approval was by no means the incitement to decide to participate in this project.	
• I understand that auditors, representatives of the client, the Medical Ethics Committee or competent authorities may wish to inspect my/my child's data in order to verify the information collected. By signing this document, I consent to this inspection.	
• I have been informed that both personal data and data concerning my/ my child's health are being processed and stored within the framework of this project. I agree with this and I am aware that I have the right to access and correct these data.	

I agree to the collection of the following body materials *(list as applicable)*:

Blood and liquid derivatives	
Genetic material (DNA, RNA) from the white blood cells	
Urine	
Tissue	
.....	

The materials will be stored as part of the project. I also agree that:

• My/my child pseudonymized samples and data are stored in [name of department], under the responsibility of [name of PI]	
• My/my child's pseudonymized data is available now and for future studies, even if I am no longer able to make decisions about it myself or after my death.	
• My/my child's administrative and medical data are kept up-to-date.	

I agree that my/my child's body material will be used in the following areas of research:

Research into [disease]	
Research on [other human diseases]	

I agree that my/my child's samples and data in the context of approved scientific studies may be transferred to:

Academic institutions	
Care institutions (e.g. hospitals)	
Commercial institutions (private companies)	

I agree that my/my child's samples and data may be used:

Within (geographical area)	
Within (continent)	
In any country with the same level of privacy protection	

It is possible that you will be contacted within the framework of this project for more information or to provide you/your child's individual findings:

I agree to be contacted again for additional information, e.g. via questionnaires.	
I do not wish to be informed of 'incidental findings' that may have an impact on my/ my child's state of health.	
I would like to be informed by my attending physician about incidental findings.	

Name and first name of participant: ………………………………………………………
Date: ………/……./……..
Signature:

<u>Taking ICF</u>:

Name and first name study coordinator/doctoral researcher: ……………………………

I declare that I have verbally provided the necessary information concerning this project (its nature, purpose, and foreseeable effects) as well as a copy of the information document to the participant.	
I confirm that no pressure has been exerted on the participant to get him/her to agree to participate in this project and I am willing to answer any additional questions you may have.	

Date: ………/……./……..
Signature:

2 copies should be completed. The original will be kept by the researcher in the hospital for [years], the copy will be given to the participant or parents or legal representatives of participants.
